# Multidimensional comprehensive and integrated analysis of the potential function of TMEM25 in renal clear cell carcinoma with low expression status

**DOI:** 10.18632/aging.205372

**Published:** 2024-01-05

**Authors:** Ping Xi, Zhicheng Zhang, Yifu Liu, Yechen Nie, Binbin Gong, Ji Liu, Hao Huang, Ziwen Liu, Ting Sun, Wenjie Xie

**Affiliations:** 1 Department of Urology, The First Affiliated Hospital of Nanchang University, Nanchang 330006, Jiangxi Province, China; 2 Department of Surgery, Fuzhou First People’s Hospital, Fuzhou 344000, Jiangxi Province, China

**Keywords:** renal clear cell carcinoma, TMEM25, immune infiltration, prognostic biomarker, DNA methylation

## Abstract

Background: Transmembrane 25(TMEM25) stands out as a potential prognostic biomarker and therapeutic target in the realm of cancer, yet its precise mechanism of action within clear cell renal cell carcinoma (ccRCC) remains unclear.

Materials and Methods: Gene expression data and clinically relevant information extracted from The Cancer Genome Atlas (TCGA) and Gene expression omnibus (GEO) databases unveil the expression patterns of TMEM25 within renal clear cell carcinoma, which reveals its prognostic and diagnostic significance. The protein expression data is available via the Human Protein Atlas (HPA) database. Further, qPCR experiments conducted on cells and tissues provide strong evidence of the gene’s expression status. Additionally, they explore the correlations between TMEM25 expression and DNA methylation, gene mutations, immune cell infiltration, and drug sensitivity within this specific tumor context.

Results: At both the RNA and protein levels, TMEM25 displays a noteworthy downregulation in expression, which is consistently linked to an unfavorable prognosis. Receiver Operating Characteristic (ROC) curve analysis, univariate and multivariate Cox regression analyses confirmed the ability of TMEM25 to diagnose and determine prognosis in ccRCC. Its expression related closely with various immune cell types, immune checkpoints, immune inhibitors, and MHC molecules. Within ccRCC tissues, TMEM25 DNA methylation levels are observed to be elevated, and this upregulation is observed across various conditions. TMEM25 mutations also have an impact on the prognosis of ccRCC patients and the results of drug sensitivity analyses are useful for clinical decision-making.

Conclusions: TMEM25 in ccRCC could potentially function as a tumor suppressor gene, holding substantial promise as a novel biomarker for diagnosing, treating, and prognosticating ccRCC patients.

## INTRODUCTION

Being the most common form of kidney cancer, renal cell carcinoma (RCC) originates from malignant changes in renal epithelial cells. It’s estimated that in 2022, approximately 79,000 people worldwide received an RCC diagnosis, with a potential 13,920 succumbing to the disease. This places a notable economic strain on the global healthcare system [1]. Some of the risk factors that contribute to the development and progression of RCC include obesity, high blood pressure, and smoking [2]. RCC is mainly composed of subtypes such as ccRCC, papillary renal cell carcinoma, and chromophobe renal cell carcinoma. Among these, ccRCC stands out, constituting 75-80% of cases. This particular subtype usually shows a higher degree of malignancy, morbidity and mortality [2–4]. Evidence indicates that due to the unique characteristics of ccRCC, it often shows limited sensitivity to radiation and chemotherapy. Consequently, surgery remains the cornerstone of treatment for ccRCC [5]. The survival rate for patients diagnosed with this condition in its early stages can reach an impressive 80-90%. However, a substantial number of patients initially exhibit no symptoms, and approximately one-third of patients are already diagnosed with distant metastases at the time of diagnosis. Unfortunately, the survival rate for patients with metastatic RCC remains less than satisfactory [6–9]. Therefore, there is an urgent need to explore new, validated biomarkers for early diagnosis and prognosis, as well as potential therapeutic targets.

Transmembrane proteins (TMEM) are a group of transmembrane proteins, and members of this family of proteins have different functions in a range of biological processes. For example, TMEM165 plays a pivotal role in Golgi glycosylation and the preservation of Golgi morphology [10]; meanwhile, TMEM97 contributes to the development and differentiation of the liver [11]. In the field of cancer genomics, TMEM116 emerges as a pivotal integrator of carcinogenic signaling in lung cancer metastasis [12]. Conversely, the reduction of TMEM45B activity inhibits gastric cancer cell proliferation through suppression of the JAK2/STAT3 pathway; and this also inhibits the proliferation, invasion and tumourigenesis of osteosarcoma cell [13, 14]; Some of these factors are also used as prognostic biomarkers, as seen in kidney cancer [15–17]. Indeed, as part of the TMEM protein family, TMEM25 is also of great importance in the field of cancer, where it plays an active role in the process of cancer development and progression. For instance, both hypermethylation and down-regulation of TMEM25 have been associated with regulating colorectal cancer development and progression [18]; In breast cancer, TMEM25 stands as a favorable indicator for prognosis and prediction [19], where its decreased expression enhances the sensitivity of MCF-7/PR cells to paclitaxel treatment [20]. These revelations collectively hint at TMEM25’s potential as both a prognostic biomarker and a target for therapeutic interventions in cancer. However, the precise mechanisms through which it operates within ccRCC are still not fully elucidated. Therefore, it is crucial to promptly investigate whether TMEM25 could potentially function as a vital prognostic biomarker and therapeutic target in the context of ccRCC.

To ascertain the prognostic and diagnostic implications of TMEM25 in ccRCC, we investigated its varying expression patterns using data from TCGA, GEO databases, and samples collected at our research center. Additionally, we explored the interplay between TMEM25 expression and factors such as DNA methylation, gene alterations, immune cell infiltration, and drug sensitivity. Concurrently, we delved into potential mechanisms that could account for its functional role.

## RESULTS

### Differential expression of TMEM25 in ccRCC tissues

**Figure 1:**
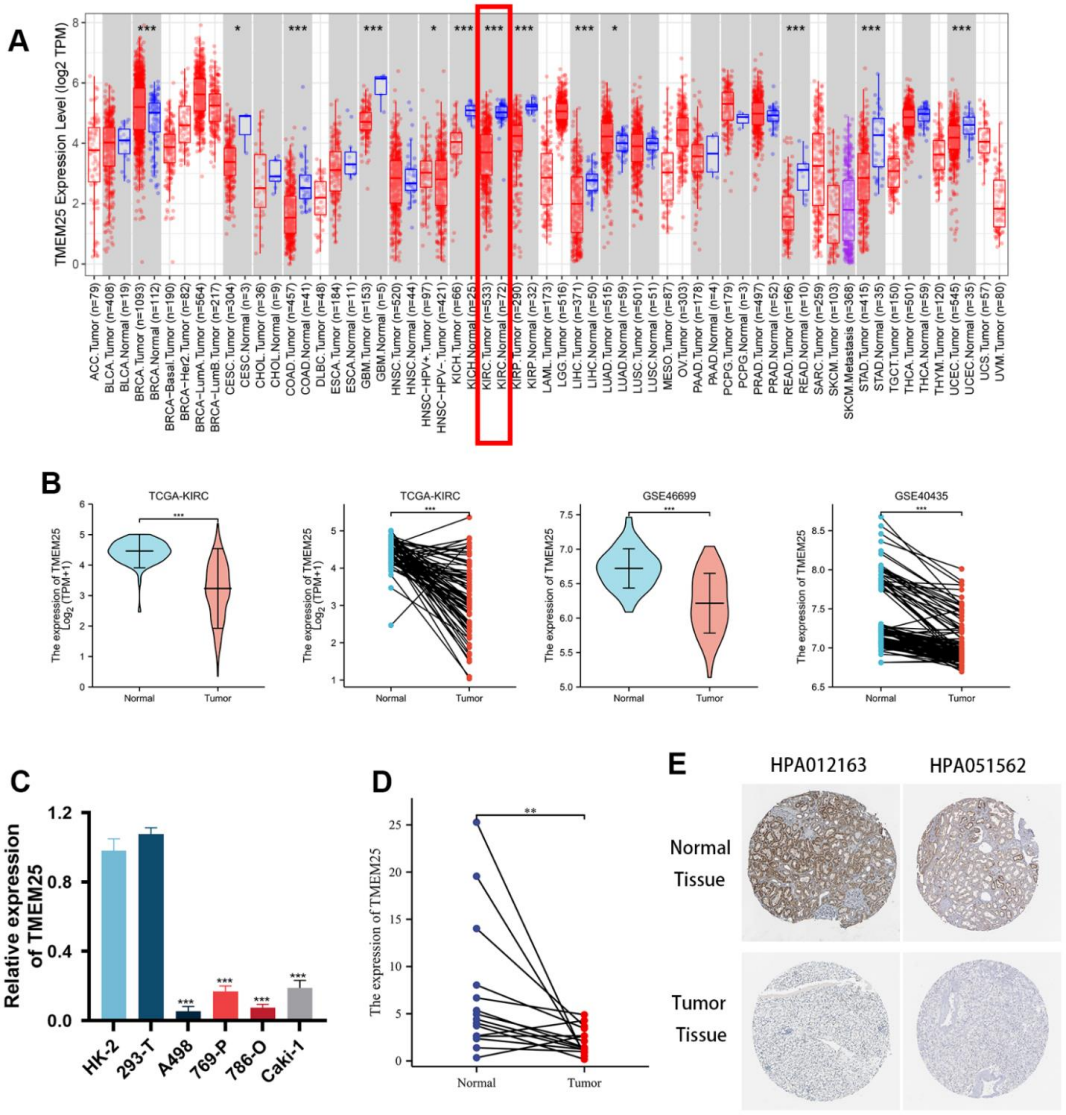
**Differential expression of RNA levels and protein levels of TMEM25 in ccRCC.** (**A**) Pan-cancer analysis showed that differential expression of TMEM25 RNA levels was present in a variety of cancers, including renal clear cell carcinoma, which showed a significant low expression status. (**B**) TCGA-KIRC, GSE46699 and GSE40435 cohort differential analysis consistently showed that TMEM25 expression was significantly lower in ccRCC than in normal tissues. (**C**) Compared with normal renal tubular epithelial cells (HK-2) and the human embryonic kidney cell line(293-T), TMEM25 was also lowly expressed in renal cancer cell lines. (**D**) The results of the samples from our study center also further confirmed that TMEM25 was expressed much less in ccRCC. (**E**) The HPA database demonstrated that TMEM25 expression in ccRCC tissues is significantly lower than that in normal tissues. (*p < 0.05, **p < 0.01, ***p < 0.001).

This study presents a comprehensive examination of TMEM25 expression across various cancers, accomplished through a pancancer analysis. The outcomes of this pan-cancer TMEM25 analysis, derived from the TIMER online platform. Specifically, TMEM25 exhibited high expression levels in breast invasive carcinoma (BRCA) and lung adenocarcinoma (LUAD). However, a noticeable reduction in expression was evident in several other cancers, including cervical squamous cell carcinoma and endocervical adenocarcinoma (CESC), colon adenocarcinoma (COAD), glioblastoma multiforme (GBM), kidney chromophobe (KICH), kidney renal clear cell carcinoma (KIRC), kidney renal papillary cell carcinoma (KIRP), liver hepatocellular carcinoma (LIHC), rectum adenocarcinoma (READ), stomach adenocarcinoma (STAD), and uterine corpus endometrial carcinoma (UCEC) tissues when compared to their respective normal tissues (Figure 1A). Within the TCGA-KIRC cohort, we conducted an in-depth analysis of TMEM25 expression, which showed a significant decrease in TMEM25 expression levels in ccRCC compared to normal tissue. This finding was confirmed in paired analyses of ccRCC and normal tissue samples (Figure 1B). Concurrently, our investigations extended to additional cohorts such as GSE46699 and GSE40435, where TMEM25 exhibited lower expression in ccRCC in contrast to normal tissues. This observation was further affirmed through qPCR analysis of renal cancer cell lines and renal clear cell carcinoma tissues (Figure 1C, 1D). Meanwhile, immunohistochemical assessment from the Human Protein Atlas (HPA) database confirmed low expression of TMEM25 in ccRCC (Figure 1E).

### Correlation analysis of TMEM25 expression and clinicopathological features

**Figure 2:**
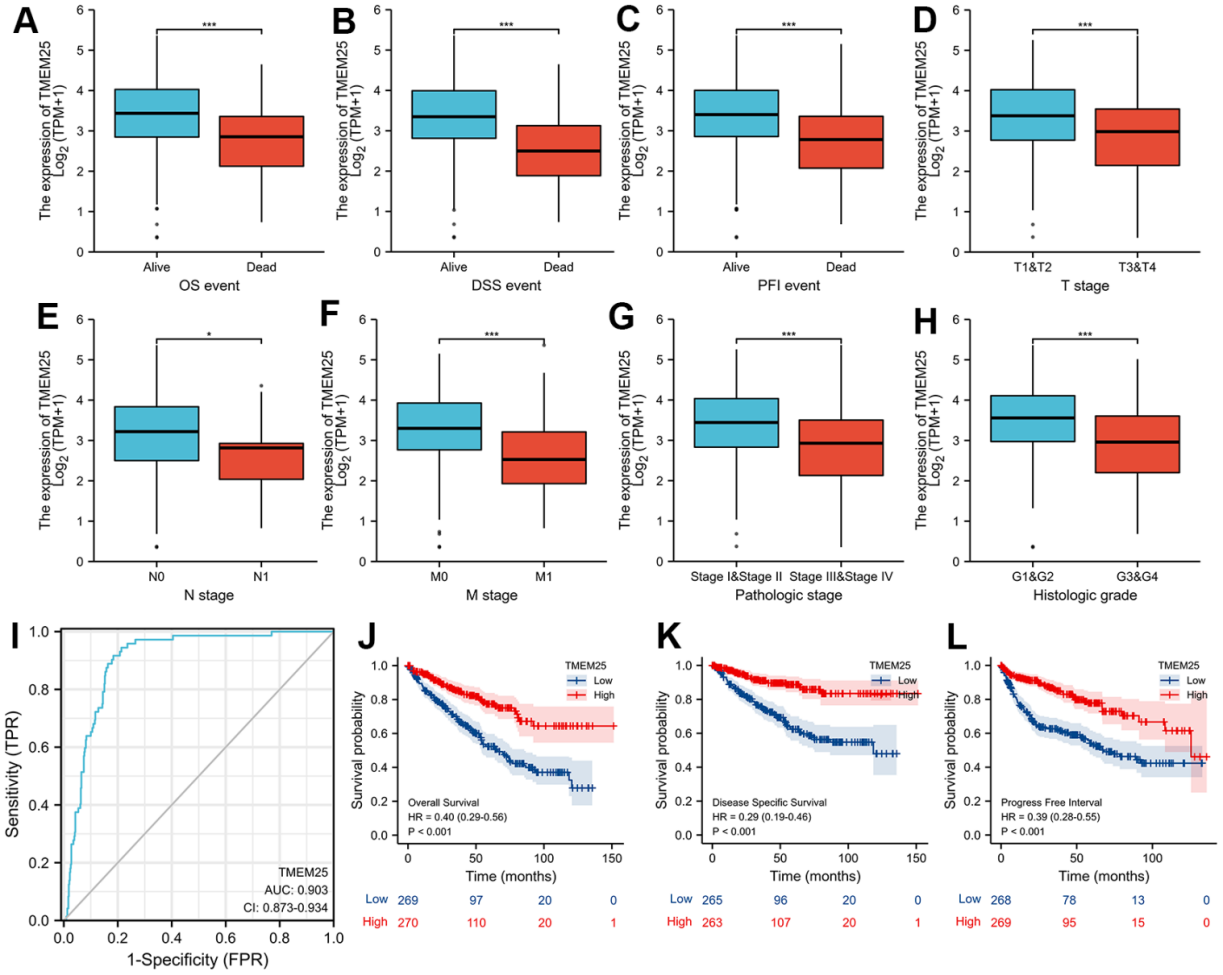
**Clinical significance of TMEM25 in ccRCC.** The differential expression of TMEM25 was analyzed in different clinical states such as OS (**A**), DSS (**B**), PFI (**C**), T stage (**D**), N stage (**E**), M stage (**F**), pathologic stage (**G**) and histologic grade (**H**). (**I**) The ROC curve demonstrated a strong ability of TMEM25 in distinguishing ccRCC patients from normal patients (AUC value of 0.903). The prognosis of ccRCC patients in the TMEM25 high expression group was significantly better than that of the low expression group in terms of OS (**J**), DSS (**K**), and PFI (**L**). (***p < 0.001).

Based on the levels of TMEM25 RNA expression within the TCGA-KIRC cohort, patients were categorized into distinct groups: those with high and low expression levels. By combining these results with clinically relevant features, we revealed noteworthy associations between TMEM25 and several factors. These encompassed age (p=0.011), gender (p=0.040), T-stage (p<0.001), N-stage (p=0.009), M-stage (p<0.001), Pathologic stage (p<0.001), and Histologic grade (p<0.001) (Table 1). In addition, our study highlights large differences in key survival factors. Specifically, overall survival (OS), disease-specific survival (DSS) and progression-free interval (PFI) exhibited significant differences between the two groups of ccRCC patients (all p-values < 0.001) (Table 1). In this study, we found that for OS, DSS, and PFI the expression of TMEM25 was lower in the presence of adverse events (for example death events). Similarly, the expression of TMEM25 was significantly lower in later T-stages, N-stages, M-stages, pathological stages and histological stages than in corresponding earlier stages (Figure 2A–2H).

**Table 1:** The correlation between TMEM25 expression level and clinicopathological factors in ccRCC.

**Characteristic**	**Low expression of TMEM25**	**High expression of TMEM25**	**p**
n	269	270	
Age, n (%)			**0.011**
<=60	119 (22.1%)	150 (27.8%)	
>60	150 (27.8%)	120 (22.3%)	
Gender, n (%)			**0.040**
Female	81 (15%)	105 (19.5%)	
Male	188 (34.9%)	165 (30.6%)	
T stage, n (%)			**< 0.001**
T1	103 (19.1%)	175 (32.5%)	
T2	42 (7.8%)	29 (5.4%)	
T3	117 (21.7%)	62 (11.5%)	
T4	7 (1.3%)	4 (0.7%)	
N stage, n (%)			**0.009**
N0	122 (47.5%)	119 (46.3%)	
N1	14 (5.4%)	2 (0.8%)	
M stage, n (%)			**< 0.001**
M0	199 (39.3%)	229 (45.3%)	
M1	58 (11.5%)	20 (4%)	
Pathologic stage, n (%)			**< 0.001**
Stage I	98 (18.3%)	174 (32.5%)	
Stage II	33 (6.2%)	26 (4.9%)	
Stage III	76 (14.2%)	47 (8.8%)	
Stage IV	60 (11.2%)	22 (4.1%)	
Histologic grade, n (%)			**< 0.001**
G1	1 (0.2%)	13 (2.4%)	
G2	90 (16.9%)	145 (27.3%)	
G3	117 (22%)	90 (16.9%)	
G4	57 (10.7%)	18 (3.4%)	
OS event, n (%)			**< 0.001**
Alive	147 (27.3%)	219 (40.6%)	
Dead	122 (22.6%)	51 (9.5%)	
DSS event, n (%)			**< 0.001**
Alive	182 (34.5%)	238 (45.1%)	
Dead	83 (15.7%)	25 (4.7%)	
PFI event, n (%)			**< 0.001**
Alive	158 (29.3%)	220 (40.8%)	
Dead	111 (20.6%)	50 (9.3%)	

### Evaluation of TMEM25 in ccRCC for diagnosis and determination of prognostic ability, and construction and validation of nomogram

We designed a Receiver Operating Characteristic (ROC) curve to evaluate the capability of TMEM25 expression in effectively distinguishing between ccRCC and the adjacent normal tissue. The outcomes showed a remarkable area under the curve (AUC) value of 0.903, strongly indicating the substantial potential of TMEM25 as a promising diagnostic marker (Figure 2I). Taking these findings further, our study proceeded to validate the predictive power of TMEM25 in determining the prognosis of ccRCC patients. Specifically, ccRCC patients exhibiting lower TMEM25 gene expression consistently experienced shorter OS, DSS, and PFI (all p-values <0.001) (Figure 2J–2L). These results have highlighted the important impact of TMEM25 expression on the prognosis of ccRCC patients.

In our univariate COX regression analysis, we observed that a range of factors, including T stage, N stage, M stage, pathologic stage, histologic stage, and TMEM25 expression had a significant effect on OS, DSS, and PFI in ccRCC patients. On the basis of these preliminary results, our subsequent multivariate COX regression analyses consistently highlighted the potential significance of TMEM25 expression. It emerged as a substantial and independent risk factor, holding promise in predicting these outcomes (Tables 2–4). This further underscores the clinical relevance of TMEM25 expression in offering valuable prognostic insights for patients with ccRCC.

**Table 2:** Univariate and multivariate Cox regression analyses of factors predicting overall survival in ccRCC.

**Characteristics**	**Total (N)**	**Univariate analysis**		**Multivariate analysis**	
		**Hazard ratio (95% CI)**	**P-value**	**Hazard ratio (95% CI)**	**P-value**
T stage	539				
T1&T2	349	Reference			
T3&T4	190	3.228 (2.382-4.374)	**<0.001**	1.391 (0.613-3.159)	0.430
N stage	257				
N0	241	Reference			
N1	16	3.453 (1.832-6.508)	**<0.001**	1.406 (0.699-2.829)	0.339
M stage	506				
M0	428	Reference			
M1	78	4.389 (3.212-5.999)	**<0.001**	2.543 (1.516-4.267)	**<0.001**
Pathologic stage	536				
Stage I&Stage II	331	Reference			
Stage III&Stage IV	205	3.946 (2.872-5.423)	**<0.001**	1.433 (0.568-3.617)	0.447
Histologic grade	531				
G1&G2	249	Reference			
G3&G4	282	2.702 (1.918-3.807)	**<0.001**	1.539 (0.924-2.563)	0.098
TMEM25	539				
Low	269	Reference			
High	270	0.403 (0.291-0.559)	**<0.001**	0.544 (0.340-0.870)	**0.011**

**Table 3:** Univariate and multivariate Cox regression analyses of factors predicting disease-specific survival in ccRCC.

**Characteristics**	**Total (N)**	**Univariate analysis**		**Multivariate analysis**	
		**Hazard ratio (95% CI)**	**P-value**	**Hazard ratio (95% CI)**	**P-value**
T stage	528				
T1&T2	346	Reference			
T3&T4	182	5.542 (3.652-8.411)	**<0.001**	1.149 (0.496-2.660)	0.745
N stage	255				
N0	240	Reference			
N1	15	3.852 (1.825-8.132)	**<0.001**	1.234 (0.564-2.698)	0.599
M stage	495				
M0	421	Reference			
M1	74	9.108 (6.209-13.361)	**<0.001**	3.578 (1.986-6.447)	**<0.001**
Pathologic stage	525				
Stage I&Stage II	328	Reference			
Stage III&Stage IV	197	9.835 (5.925-16.325)	**<0.001**	3.318 (1.112-9.905)	**0.032**
Histologic grade	520				
G1&G2	248	Reference			
G3&G4	272	4.793 (2.889-7.952)	**<0.001**	1.682 (0.837-3.377)	0.144
TMEM25	528				
Low	265	Reference			
High	263	0.291 (0.186-0.455)	**<0.001**	0.479 (0.259-0.885)	**0.019**

**Table 4:** Univariate and multivariate Cox regression analyses of factors predicting progression-free intervals in ccRCC.

**Characteristics**	**Total (N)**	**Univariate analysis**		**Multivariate analysis**	
		**Hazard ratio (95% CI)**	**P-value**	**Hazard ratio (95% CI)**	**P-value**
T stage	537				
T1&T2	349	Reference			
T3&T4	188	4.522 (3.271-6.253)	**<0.001**	1.041 (0.517-2.098)	0.911
N stage	256				
N0	240	Reference			
N1	16	3.682 (1.891-7.167)	**<0.001**	1.069 (0.524-2.180)	0.854
M stage	504				
M0	428	Reference			
M1	76	8.968 (6.464-12.442)	**<0.001**	4.295 (2.518-7.327)	**<0.001**
Pathologic stage	534				
Stage I&Stage II	331	Reference			
Stage III&Stage IV	203	6.817 (4.770-9.744)	**<0.001**	3.431 (1.415-8.317)	**0.006**
Histologic grade	529				
G1&G2	249	Reference			
G3&G4	280	3.646 (2.503-5.310)	**<0.001**	1.500 (0.886-2.539)	0.131
TMEM25	537				
Low	268	Reference			
High	269	0.391 (0.279-0.548)	**<0.001**	0.566 (0.345-0.929)	**0.024**

**Figure 3:**
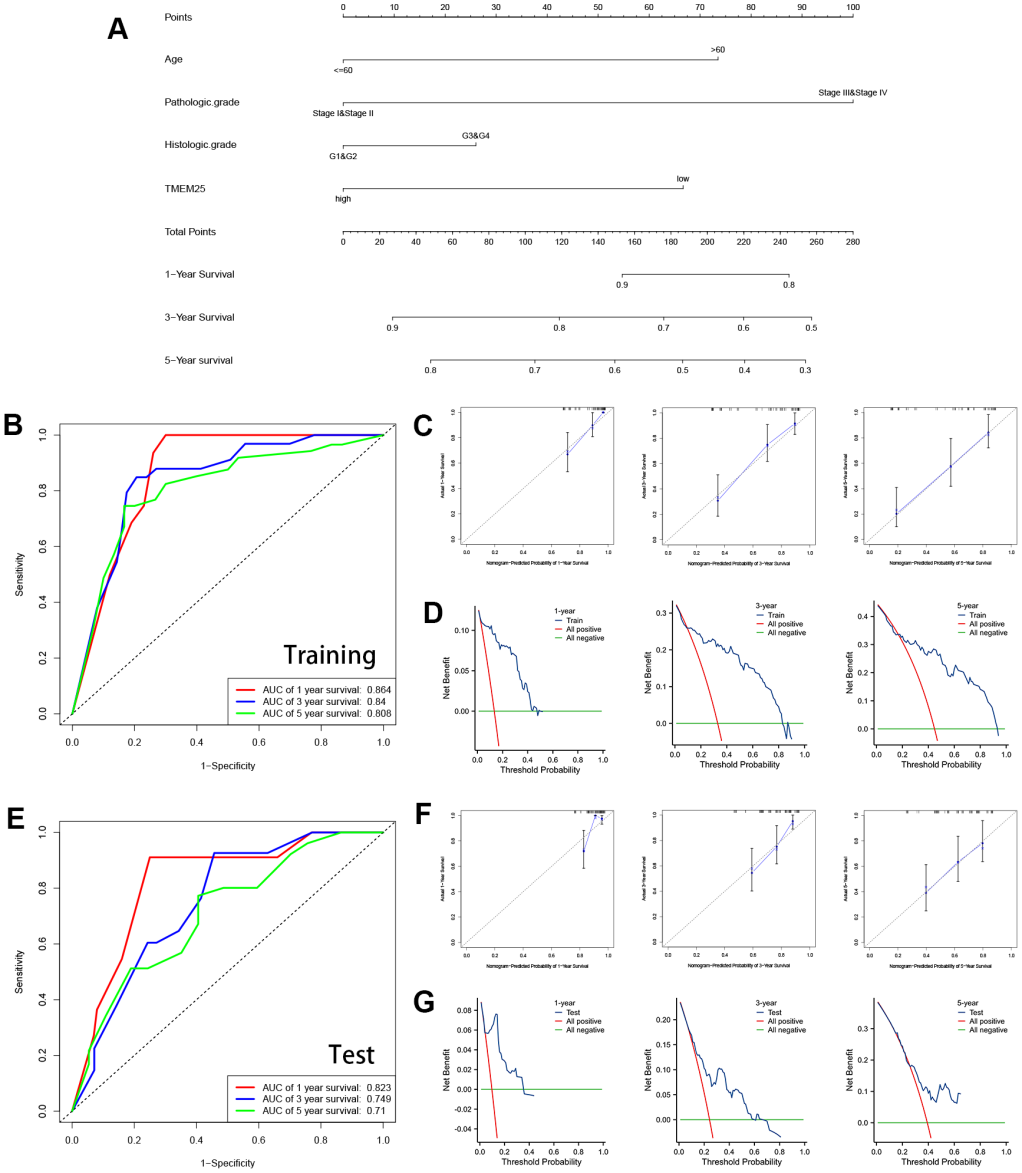
**Construction and validation of nomograms containing TMEM25 expression data.** (**A**) Nomograms containing age, pathologic grade, histologic grade and TMEM25 expression were constructed. The TCGA-KIRC cohort was randomized into TRAINING and TEST groups to mutually validate the ability of the model in combination with the survival prognosis of the patients. (**B**) In the training group, the ROC curves demonstrated AUC values of 0.864, 0.84, and 0.808 for predicting survival at 1, 3, and 5 years, respectively; the calibration plots (**C**) and clinical decision curves (**D**) at 1, 3, and 5 years were consistent in indicating that the model had strong predictive power. (**E**) Similarly, in the test group, the ROC curves demonstrated AUC values of 0.823, 0.749, and 0.71 for predicting survival at 1, 3, and 5 years, respectively; the results of the calibration plots (**F**) and clinical decision curves (**G**) at 1, 3, and 5 years were consistent with the training group.

These findings underscore the potential significance of TMEM25 as a prognostic factor within the context of ccRCC. Expanding on the diagnostic and prognostic capabilities of TMEM25 in ccRCC, we developed a comprehensive nomogram that integrates some variables including age, pathologic grade, histologic grade and TMEM25 expression. ROC curves calculated from the nomogram in the training group (Figure 3A) yielded impressive AUC values of 0.864, 0.84, and 0.808 at 1, 3, and 5 years respectively (Figure 3B). These robust AUC values reflect the nomogram’s strong predictive power. Additionally, calibration curves and clinical decision curves further affirmed the nomogram’s effectiveness, showing that the predictions of the nomograms were in perfect agreement with the actual survival trends of the patients (Figure 3C, 3D). Furthermore, the AUC values for the ROC curves in the test group at 1, 3, and 5 years were 0.823, 0.749, and 0.71 respectively (Figure 3E), highlighting a consistent correlation between the calibration curves and the clinical decision curve (Figure 3F, 3G). In summary, the inclusion of TMEM25 in the nomogram offers a valuable and practical tool for predicting the survival probabilities of patients with ccRCC.

### Discovery of potential functions of TMEM25 in ccRCC

**Figure 4:**
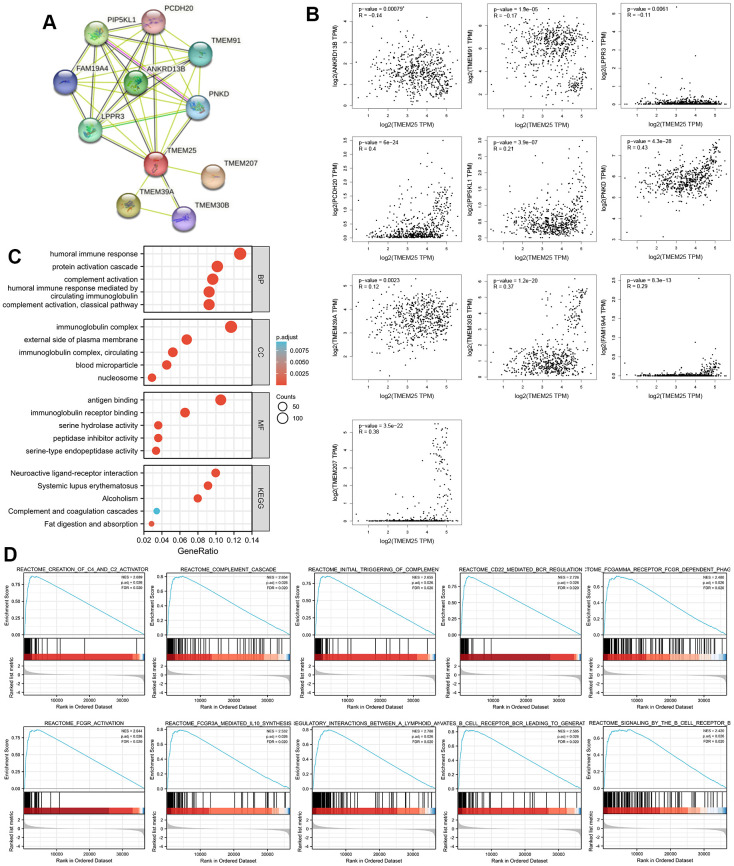
**Enrichment analysis of the potential functions played by TMEM25 in ccRCC.** (**A**) The STRING website analyzes and maps protein-protein interaction networks of genes that have some association with TMEM25. (**B**) The GEPIA2 website further analyzed the correlation of TMEM25 with the expression of these protein-protein interacting genes in ccRCC. (**C**) GO, KEGG pathway enrichment analysis reveals potential function of TMEM25 in ccRCC. (**D**) GSEA enrichment analysis further explored its potential function.

By using the capabilities of the STRING database, we were able to construct a protein-protein interaction network centered around TMEM25. This notable set of genes includes ANKRD13B, TMEM91, LPPR3, PCDH20, PIP5KL1, PNKD, TMEM39A, TMEM30B, FAM19A4, and TMEM207 (Figure 4A). Subsequent to this network construction, we embarked on a Spearman analysis to unravel the correlation between these ten genes and TMEM25. This analysis was conducted using TCGA-KIRC tumor and normal tissues, with the help of the GEPIA2 website. The outcomes of this study yielded valuable results. We observed a distinctive pattern where the expression levels of TMEM25 exhibited a negative correlation with ANKRD13B, TMEM91, and LPPR3. In contrast, the expression levels of PCDH20, PIP5KL1, PNKD, TMEM39A, TMEM30B, FAM19A4, and TMEM207 displayed a positive correlation with TMEM25 expression (Figure 4B). This complex web of correlations deepens our understanding of TMEM25’s potential functional roles within the context of ccRCC.

We carried out functional enrichment analyses based on 178 up-regulated genes and 4365 down-regulated genes identified in the TCGA-KIRC dataset from the differential analysis of the TMEM25 high and low expression groups. Our exploration revealed intriguing insights into the potential roles of TMEM25 within ccRCC. Regarding the Gene Ontology (GO) results, it was apparent that TMEM25 could be intricately associated with an array of biological processes (BP). These processes encompassed “humoral immune response,” “protein activation cascade,” “complement activation,” and “humoral immune response mediated by circulating immunoglobulin.” In terms of cell components (CC), TMEM25’s influence extended to structures like “immunoglobulin complex,” “external side of plasma membrane,” “immunoglobulin complex, circulating,” “blood microparticle,” and “nucleosome.” Moving on to molecular functions (MF), the enrichment was evident in areas such as “antigen binding,” “immunoglobulin receptor binding,” “serine hydrolase activity,” “peptidase inhibitor activity,” and “serine-type endopeptidase activity.” Parallel to these results, the KEGG enrichment results highlighted significant pathways. These pathways encompassed “neuroactive ligand-receptor interaction,” “systemic lupus erythematosus,” “alcoholism,” “complement and coagulation cascades,” and “fat digestion and absorption” (Figure 4C).

Furthermore, through Gene Set Enrichment Analysis (GSEA), we uncovered a series of enriched gene sets linked with TMEM25 in ccRCC. These sets included “complement cascade,” “creation of C4 and C2 activators,” “initial triggering of complement,” “CD22-mediated BCR regulation,” “FCGAMMA receptor FCGR-dependent phagocytosis,” “FCGR activation,” “FCGR3A-mediated IL10 synthesis,” “immunoregulatory interactions between a lymphoid and a non-lymphoid cell,” “antigen activates B cell receptor BCR leading to generation of second messengers,” and “signaling by the B cell receptor BCR” (Figure 4D). These comprehensive findings collectively offer valuable insights into the potential functional roles of TMEM25 within complexities of ccRCC.

### The relationship between TMEM25 expression and ccRCC immune infiltration

**Figure 5:**
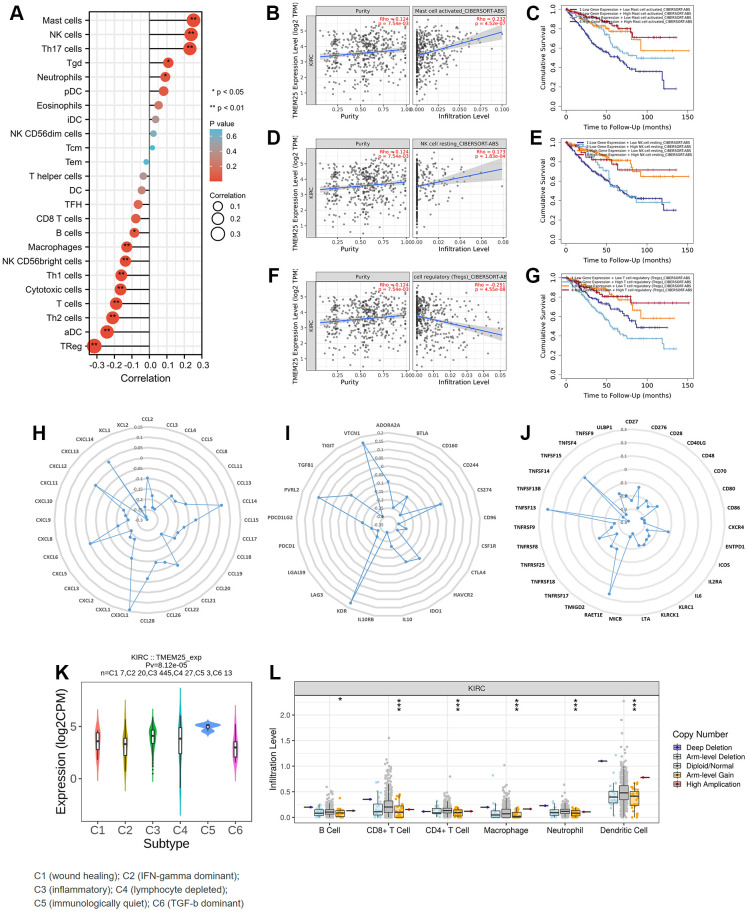
**Immune infiltration correlation analysis of TMEM25 in ccRCC.** (**A**) The ssGSEA algorithm analyzes the correlation between TMEM25 and various immune cell infiltrations in ccRCC. (**B**–**G**) The TIMER database used the cibersort-abs algorithm to calculate the correlation between TMEM25 expression and mast cell activated, NK cell resting, and Tregs in ccRCC, and plotted the Kaplan-Meier curves in relation to their prognosis. (**H**–**J**) A more comprehensive analysis conducted by TISIDB showed that TMEM25 expression in ccRCC is strongly associated with a variety of immune checkpoints, immunosuppressants, and MHC molecules. (**K**, **L**) The TISIDB website analyzed the correlation between TMEM25 expression in ccRCC and immune subtypes, and examined the relationship between its copy number and immune infiltrating cells. (*p < 0.05, **p < 0.01, ***p < 0.001).

In the context of functional enrichment analysis, it was revealed that TMEM25 might possess immunological significance in ccRCC. Subsequent ssGSEA calculations demonstrated that the expression of TMEM25 correlated both positively and negatively with immune infiltration. Specifically, it was positively correlated with Treg (regulatory T cells) infiltration and negatively correlated with resting NK (natural killer) cell infiltration, as well as with NK cell infiltration overall (Figure 5A). Survival analysis conducted using data from the TIMER2.0 website suggested that ccRCC patients exhibiting low TMEM25 expression, along with reduced infiltration of activated and resting mast cells, as well as resting NK cells, and elevated infiltration of Tregs, were associated with poorer clinical outcomes (Figure 5B–5G). Further comprehensive analysis carried out using the TISIDB platform indicated a strong association between TMEM25 expression in ccRCC and a range of immune checkpoints, immunosuppressive agents, and major histocompatibility complex (MHC) molecules (Figure 5H–5J).

In addition, the TISIDB analysis also explored the relationship between TMEM25 expression in ccRCC and distinct immune subtypes. This analysis revealed that TMEM25 expression was most statistically significant in ccRCC subtype C5 (characterized as immunologically quiet) and lowest in ccRCC subtype C6 (dominated by TGF-b signaling). This implies that TMEM25 expression in the ccRCC immunomicroenvironment has a significant impact on the immune environment to some extent (Figure 5K). Another aspect of investigation involved examining the connection between TMEM25 copy numbers and the presence of immune infiltrating cells. The results indicated that higher TMEM25 levels associated with arm-level gain status were inversely correlated with the presence of B cells, CD8+ T cells, CD4+ T cells, macrophages, neutrophils, and dendritic cells, in contrast to the diploid/normal state. This suggests a significant relationship between TMEM25 copy number and the extent of immune infiltration in ccRCC (Figure 5L).

### DNA methylation analysis of TMEM25 in ccRCC patients

**Figure 6:**
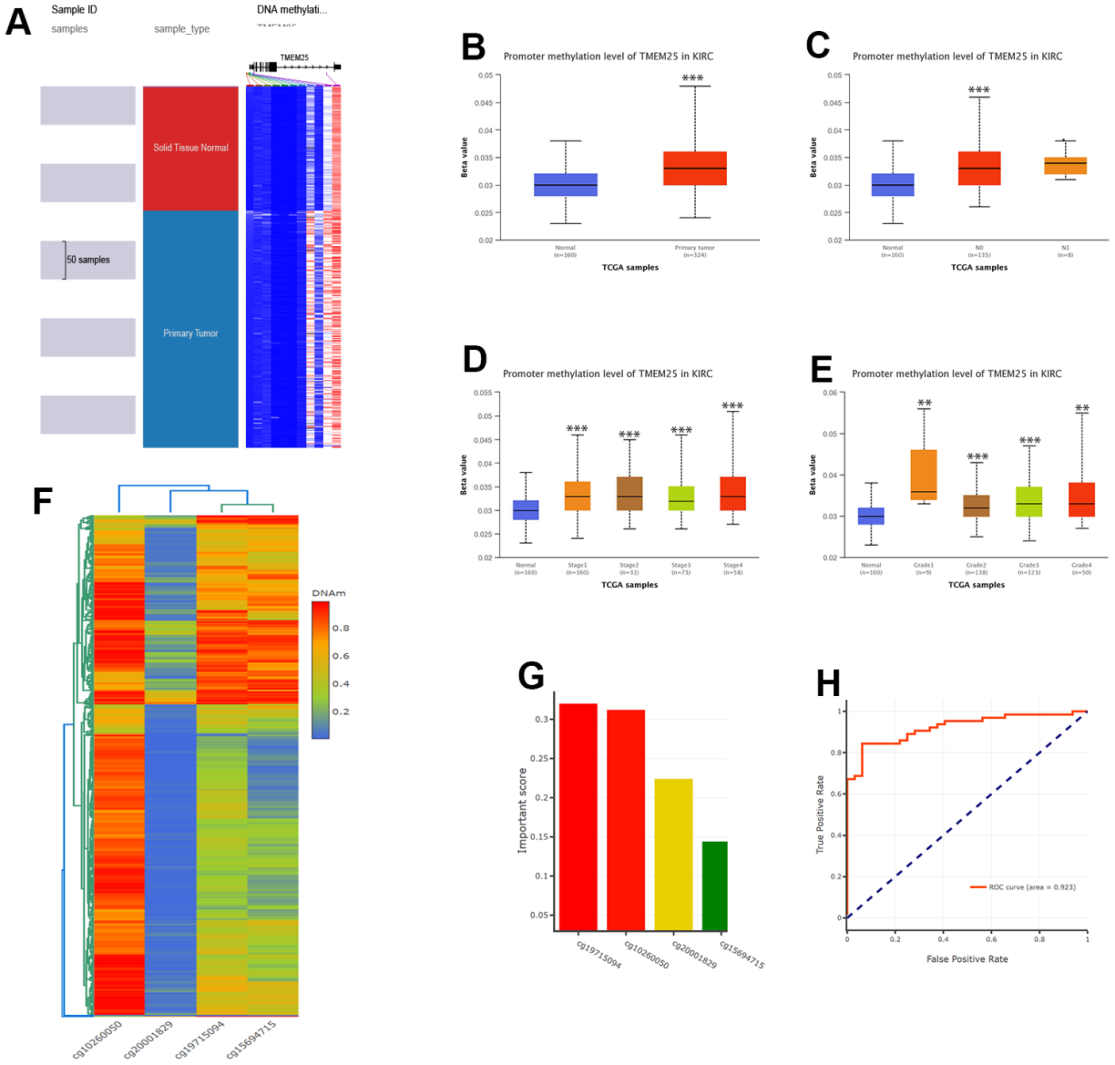
**DNA methylation analysis of TMEM25 in ccRCC patients.** (**A**–**E**) UCSC Xena and UALCAN websites revealed for us the TMEM25 DNA methylation expression levels in ccRCC in different states. (**F**, **G**) The DNMIVD database further demonstrated that the CpG loci in TMEM25 was associated with the diagnosis and prognosis of ccRCC patients and screened for an important role of the four CpG islands. (**H**) The ROC curves calculated from the diagnostic model consisting of these four CpG loci showed a strong ability to distinguish ccRCC from normal tissue. (**p < 0.01, ***p < 0.001).

Upon conducting a thorough analysis of TMEM25 DNA methylation data using the UCSC Xena and UALCAN platforms, compelling findings have come to explore. The investigation has unveiled a significant increase in DNA methylation levels within the TMEM25 gene in ccRCC tissues compared to their normal tissues. This elevated methylation trend was consistently observed not only in cases with nodal metastasis but also across varying cancer stages and diverse tumor grades (Figure 6A–6E). Further exploration into the DNMIVD database has yielded deeper insights. The analysis has revealed distinct associations between specific CpG loci within the TMEM25 gene and both the diagnosis and prognosis of ccRCC patients. Notably, four particular CpG loci (cg19715094, cg10260050, cg20001829, cg15694715) have emerged as especially noteworthy. Among these, cg19715094 has been assigned the highest importance score, whereas cg15694715 has received the lowest importance score (Figure 6F, 6G).

The resulting diagnostic model, formed by using the information from these four identified loci, has demonstrated remarkable efficacy in discerning ccRCC tissue from normal tissue. This is evident from the ROC curves, where the diagnostic model’s ROC curve boasts a substantial AUC value of 0.923, showing its significant diagnostic capability (Figure 6H).

### Analysis of genetic alterations of TMEM25 in ccRCC

**Figure 7:**
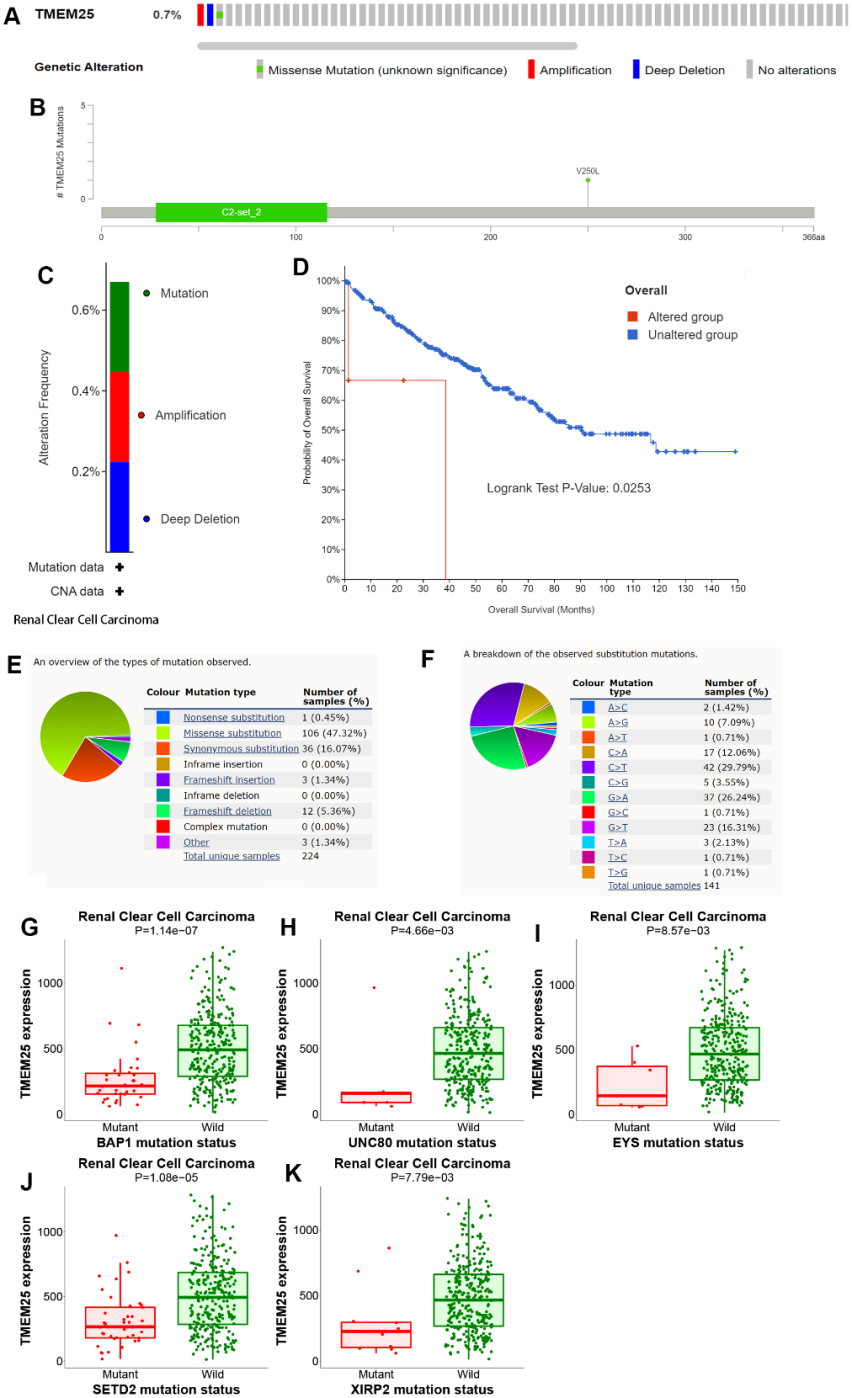
**Analysis of genetic alterations of TMEM25 in ccRCC.** (**A**–**C**) OncoPrint provides a comprehensive view of the TMEM25 mutation. (**D**) The Kaplan-Meier curves indicate differences in overall survival time between the TMEM25 mutated and unmutated groups. (**E**, **F**) The COSMIC website provides information about the types of TMEM25 gene mutations found in different types of cancer. (**G**–**K**) Mutations in BAP1, EYS, SETD2, UNC80 and XIRP2 genes alter the expression level of TMEM25.

The cBioPortal website provides a comprehensive dataset encompassing 538 ccRCC patients from the TCGA, particularly through the Firehose Legacy project. This dataset includes essential information about genetic alterations and prognostic survival outcomes. Within this cohort, a relatively small percentage (0.7%) of ccRCC patients exhibit mutations in the TMEM25 gene. These mutations primarily manifest as missense mutations and amplifications. Notably, the patients with TMEM25-altered genomes exhibit significantly lower overall survival rates in comparison to those with unaltered genomes, a difference that holds statistical significance (p=0.0253). These trends are effectively visualized in Figure 7A–7D. COSMIC website shows the types of mutations in TMEM25 in different cancers. Missense substitutions and synonymous substitutions are main mutation types, with C>T and G>A mutations emerging as the most common substitution alterations (Figure 7E, 7F). Employing the muTarget website for mutation status and TMEM25 expression analysis has uncovered an intriguing correlation. It’s evident that TMEM25 expression experiences a notable reduction in the presence of mutant phenotypes linked to genes like BAP1, EYS, SETD2, UNC80, and XIRP2 (Figure 7G–7K). This analysis highlights the intricate interactions between TMEM25 mutations and the expression profiles of these related genes.

In summary, these careful analyses performed on different platforms have combined to greatly clarify the genetic landscape shaped by mutations in the TMEM25 gene in ccRCC. Moreover, it underscores their impact on patient survival rates and their interconnectedness with genes of significance.

### Drug sensitivity analysis of TMEM25 in ccRCC

Based on an assessment of the median expression levels of TMEM25 in ccRCC, the TCGA-KIRC cohort was divided into two distinct groups: those exhibiting high TMEM25 expression and those with low TMEM25 expression. Subsequent calculations involved determining the IC50 values for a variety of drugs. A compelling trend is that ccRCC cases with lower levels of TMEM25 expression have correspondingly lower IC50 values across the range of drugs tested. Notably, this sensitivity was observed for drugs like sunitinib, pazopanib, gemcitabine, crizotinib, bryostatin, epothilone, and 5-fluorouracil. This intriguing correlation suggests that these medications tend to be more effective against ccRCC instances characterized by diminished TMEM25 expression.

**Figure 8:**
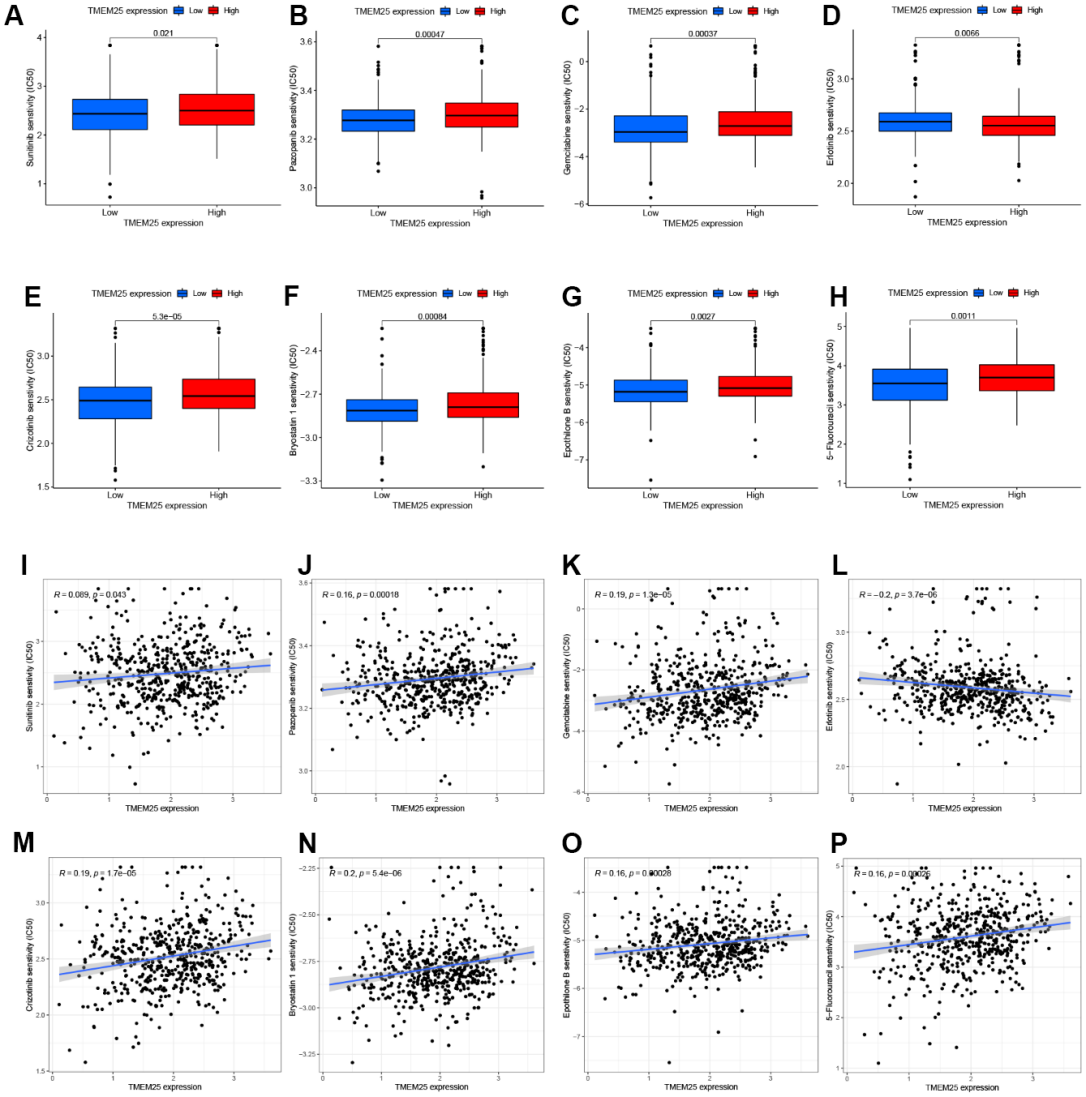
**Drug sensitivity analysis of TMEM25 in ccRCC.** Based on the median expression value of TMEM25 in ccRCC, the TCGA-KIRC cohort was categorized into high and low expression groups and the respective IC50 values were calculated, and the drug sensitivity was demonstrated by bar charts (**A**–**H**) and scatter plots (**I**–**P**).

However, an interesting divergence from this pattern is evident in the case of erlotinib. Here, ccRCC patients featuring low TMEM25 expression exhibited a lowered sensitivity to erlotinib compared to those with elevated TMEM25 expression (Figure 8A–8P). This nuanced observation highlights the multifaceted role that TMEM25 expression plays in influencing ccRCC’s response to a range of therapeutic agents. This analysis radically reveals the complex interactions between TMEM25 expression levels and drug sensitivity in ccRCC. The findings garnered from this exploration potentially carry implications for the development of personalized treatment strategies tailored to individual patients.

## DISCUSSION

While scientific and technological advancements, coupled with progress in surgical techniques, have led to certain improvements in the treatment of RCC, particularly advanced ccRCC, patients in the advanced stages of this disease still confront a formidable prognosis. In light of this, it becomes paramount to establish accurate diagnostic and predictive tools to assess the prognosis of individuals dealing with ccRCC. The genomic alignment of TMEM25 within the 11q23.3 region underscores its potential relevance within oncological and neurological contexts. Its presence in regions associated with MLL amplification and neuroblastoma deletions implies possible roles in these conditions. Additionally, the diverse tissue expression pattern of TMEM25, including both healthy and pathological contexts, suggests its involvement in processes across multiple physiological systems, with a particularly strong presence in the brain [21]. Several studies have shown that TMEM25 is involved in the regulation of tumourigenesis and progression, and in the present study, we found that TMEM25 may be acting as an oncogene in ccRCC, which is a new biomarker with great potential in the diagnosis and prognosis of ccRCC.

This revelation underscores the intricate interplay between TMEM25 and these genes, potentially highlighting shared molecular pathways or biological processes relevant to cancer. The fact that these genes are associated with different cancer types further implies their broader roles in tumorigenesis and disease progression. The high expression of ANKRD13B in ccRCC could be used with the other four genes to construct a prognostic model to predict the prognosis of the disease [22]. In HCC, activation of PCDH20 inhibited Akt and Erk activity and promoted GSK-3β signaling activity to inhibit the Wnt/β-linked protein signaling pathway [23].

Enrichment analysis has unveiled significant connections between TMEM25 and immune-related processes, further solidifying its relevance in the immunological context of ccRCC. GSEA, a powerful method for analyzing gene expression data, has provided additional insights by demonstrating the enrichment of gene sets associated with immune pathways and functions in relation to TMEM25 expression. Understanding the complexities of immune infiltration within ccRCC is crucial for understanding the underlying mechanisms that contribute to tumor growth, invasion, and potential responses to treatments. This result has the potential to guide the development of novel therapeutic strategies, including immunotherapies, that harness the power of the immune system to combat ccRCC effectively [24–26]. Our analysis indicated that the level of multiple immune cell infiltration in ccRCC was significantly correlated with TMEM25 expression. Moreover, the insights gained through further investigation using the TIMER online platform validate and extend the understanding of the relationship between TMEM25 and immunity in ccRCC. The positive correlation between TMEM25 expression and mast cell activation, along with the resting state of NK cells, as well as the negative correlation with Treg cells, underscores the dynamic interactions between TMEM25 and distinct immune cell populations. Importantly, this relationship was confirmed by survival analyses, adding greater credibility to the observed associations. These collective findings provide valuable insights into the potential mechanisms through which TMEM25 might influence the immune landscape within ccRCC. These findings contribute to the development of targeted and immunotherapies for ccRCC disease. Chemokines, immune suppressors, and immune stimulators are assuming progressively significant roles within the landscape of tumors. They are emerging as potential therapeutic targets and prognostic biomarkers for a wide array of cancers, including ccRCC [27–29]. Our findings have revealed a noteworthy observation regarding TMEM25 expression in ccRCC, demonstrating a substantial and inverse correlation with CCL5, PD-1, CTLA-4, LAG3, TIGIT, and CD80. Moreover, existing evidence suggests a compelling role for CCL5, as it has the potential to trigger the PI3K/AKT pathway, fostering epithelial-mesenchymal transition (EMT), cellular migration, and the metastatic processes within ccRCC [30]. PD-1, CTLA-4, LAG3, and TIGIT are very common immunosuppressant sites that have proven to be very beneficial in clinical practices in patients with clinically advanced ccRCC [31–34]. An intriguing revelation is that our findings have demonstrated a compelling link between lower TMEM25 expression and heightened sensitivity to a range of chemotherapy and immunotherapeutic agents. Notably, this sensitivity is particularly evident with well-known drugs for the treatment of ccRCC (such as Sunitinib and Pazopanib). This discovery holds promising potential to serve as a valuable guide for refining clinical treatment strategies [35–37].

DNA methylation is a commonly occurring heritable epigenetic modification that is damaged in all types of cancer [38, 39]. Within ccRCC, specific genes undergoing DNA hypermethylation contribute to the promotion of cancer growth and metastasis. Notably, this includes the downregulation of lncRNA ZNF582-AS1 and lncRNA APCDD1L-AS1, both of which exert regulatory control over the progression of ccRCC [40, 41]. In colorectal cancer, elevated methylation of TMEM25 exhibits an inverse correlation with its expression [18]. Likewise, our research has revealed a noteworthy finding: the levels of TMEM25 methylation within ccRCC tissues are markedly higher compared to normal tissues. This distinction in methylation patterns might hold implications linked to tumor grading and staging. Upon further investigation of CpG sites within TMEM25, it became evident that four distinct CpG sites hold significant diagnostic capabilities for ccRCC. However, the correlation between TMEM25 expression and methylation necessitates more comprehensive validation to deepen our understanding.

Genetic mutations have been a constant presence within the human body, and their aberrations and accumulation often underlie the emergence of cancer and aging [42]. A growing body of evidence supports the notion that an expanding array of genetic mutations is intricately associated with the development and progression of ccRCC [43, 44]. Based on the analysis, it’s evident that TMEM25 mutations within ccRCC predominantly encompass three distinct forms: mutation, amplification, and deep deletion. Remarkably, ccRCC patients carrying these gene mutations experience a notably reduced overall survival rate in comparison to those without these genetic alterations. Out of the 224 cancer samples, missense substitutions emerged as the predominant type of TMEM25 mutation, constituting a significant portion of 106 cases (47.32%). Notably, among the spectrum of substitution mutations, the C>T type stood out as the most prevalent. A significant reduction in TMEM25 expression was observed among the mutation groups of BAP1, UNC80, EYS, SETD2 and XIRP, compared with the wild group, as BAP1 and SETD2 are commonly occurring mutation types in kidney cancer and have poor prognosis. [43–46]. These findings provide a deeper understanding of the possible complex link between TMEM25 mutations and the development and progression of ccRCC.

We undertook a thorough exploration of TMEM25’s expression and potential roles in ccRCC, delving into various facets such as RNA and protein expression levels, pathway enrichment analysis, immune infiltration, methylation, and genetic mutations. However, it is crucial to recognise that our study has some limitations. First, our analyses are based on publicly available data, which is a limitation in itself. Second, this study did not differentially analyse other molecules of the TMEM family in ccRCC. Finally, the prediction of the functional impact of TMEM25 in ccRCC relies on bioinformatics predictions without corresponding experimental validation.

## CONCLUSIONS

In summary, our study suggests that TMEM25 might serve as a potential tumor suppressor gene in ccRCC, with reduced TMEM25 expression correlating to an adverse prognosis. In the development of ccRCC, TMEM25 appears to play a role in immune infiltration, DNA methylation and gene mutation. Consequently, TMEM25 holds significant promise as a novel biomarker for diagnosing, treating, and prognostically assessing ccRCC patients.

## MATERIALS AND METHODS

### Patient dataset acquisition

We leveraged the TCGA database (https://portal.gdc.cancer.gov/), an extensive genome sequencing repository encompassing genetic data from 33 different cancer types. From this resource, we extracted RNA expression profiles and relevant clinical data originating from 539 ccRCC samples, alongside 72 normal tissues adjacent to cancer. Furthermore, we turned to the GEO database [47] (https://www.ncbi.nlm.nih.gov/geo/) for validation purposes, utilizing GSE40435 [48] (101 tumor tissues, 101 normal tissues, platform GPL10558) and GSE46699 [49] (65 tumor tissues, 65 normal tissues, platform GPL570). The GEO database compiles gene expression data contributed by research entities globally, providing a reliable source for verifying gene expression disparities. To enhance the robustness of our findings, we procured an additional set of 15 ccRCC tumor tissue samples along with corresponding adjacent normal tissue samples at our research center. We adhered to strict ethical guidelines and all samples were ethically approved by the ethics committee of the research centre and patient approval was obtained for sample collection. Further corroborating our observations, we accessed the HPA database [50] (https://www.proteinatlas.org/), which contains comprehensive information on the tissue and cells of various human proteins. This database validated the divergent distribution patterns of TMEM25 in tumor tissues versus normal tissues of ccRCC patients.

### Cell lines and cell culture

The qPCR experiments involved normal renal epithelial cells (HK-2), the human embryonic kidney cell line (293-T), and a renal carcinoma cell line (A498, 769-P, 786-O and Caki-1) procured from the Chinese Academy of Sciences (Shanghai, China). All cell types were cultured under suitable conditions, utilizing a medium supplemented with 10% fetal bovine serum and 1% streptomycin and penicillin. The incubation temperature was maintained at 37° C with 5% CO2 to create an optimal growth environment.

### Analysis of the correlation between TMEM25 and clinicopathological features and its diagnostic and prognostic power for ccRCC

Using the RNA expression data extracted from the TCGA database and clinically relevant information, we classified the TCGA samples into different groups based on clinicopathological characteristics. Subsequently, we employed the “pROC” package within the R programming language to assess the diagnostic potential of TMEM25 in ccRCC. Furthermore, we utilized the “ggplot2”, “survival”, and “survminer” R packages to scrutinize the differential expression of TMEM25 in ccRCC across various clinical contexts. This comprehensive analysis also enabled us to determine whether such variations in expression were correlated with patient prognoses.

### Quantitative real-time polymerase chain reaction(qPCR)

Utilizing TRIzol reagent (Cwbio, Taizhou, China), we extracted RNA from both cells and tissues. The extracted RNA was then quantified using NanoDrop2000 software. Subsequently, we performed reverse transcription using reagents from TransGen Biotech ( Beijing, China). For the PCR analysis, we employed SYBR Real-Time PCR reagent from the same source (TransGen Biotech, Beijing, China), and the examination was conducted utilizing the 2^-ΔΔCt method, with β-actin serving as the internal reference.

The forward primer sequence for TMEM25 was 5′-CTTGGCACACAACCTCTCGGTG-3′, and the reverse sequence was 5′-AAGGCAAGTCCTCCAGCCACAA-3’. The reference gene was β-actin, in which the forward sequence was 5′-TCTCCCAAGTCCACACAGG-3′ and the reverse sequence was 5′-GGCACGAAGGCTCATCA-3’.

### Univariate and multivariate COX regression analyses and construction and validation of nomograms

To further validate the independent prognostic significance of TMEM25 in ccRCC, we partitioned patients within the KIRC dataset into two groups: TMEM25 high expression and TMEM25 low expression, based on the median TMEM25 expression values. Our initial step involved selecting OS, DSS, and PFI as dependent variables. Subsequently, we conducted univariate and multivariate COX regression analyses, incorporating TMEM25 and significant clinical characteristics. This aimed to ascertain whether TMEM25 stood as an independent prognostic factor, calculated by determining the corresponding Hazard Ratios (HR) and the two-sided p-values within a 95% confidence interval. Proceeding to the second step, we constructed a nomogram using the R package “rms”. This nomogram allowed us to predict the overall survival at 1, 3, and 5 years for ccRCC patients, utilizing the data derived from the previous analysis. The final step encompassed the validation of the nomogram. To achieve this, we randomly partitioned the TCGA-KIRC cohort into training and test groups using the “Caret” package. Subsequently, we evaluated the discrimination performance of the nomograms and their consistency with actual observations by means of the C-index and the calibration graphs, respectively. Additionally, we plotted DCA curves for both the training and test groups to determine the clinical utility of the nomogram.

### Enrichment analysis of protein-protein interaction network and functional pathway of TMEM25 in ccRCC

A protein-protein interaction network was established using the STRING database, wherein the combined interaction score for TMEM25 exceeded 0.4. The GEPIA2 website was employed to analyze the correlation between TMEM25 and these identified genes using the pearson correlation method. We used the “DESeq2” R package to detect gene expression differences between high and low TMEM25 expression samples in ccRCC and screened for differential genes based on p-values as well as logFC values. To comprehensively understand the functions of TMEM25 in ccRCC, we conducted an in-depth assessment. This involved analyzing GO, KEGG, and GSEA data, with BP, CC and MF corrections applied. The R packages “clusterProfiler” and “org.Hs.eg.db” were pivotal in these analyses. For GSEA, the reference gene set utilized was c2.cp.v7.2.symbols.gmt, with a seed number of 2020 and 1000 permutations for calculations.

### Analysis of immune infiltration correlation of TMEM25 in ccRCC

By employing the “GSVA” R package, we investigated the distribution and relationship of TMEM25 with immune infiltration enrichment across diverse immune cell types in ccRCC patients. This exploration encompassed the utilization of both the ssGSEA and Spearman methods. Extending our inquiry, we investigated how TMEM25 expression in ccRCC correlated with specific immune cell states. Employing the CIBERSORT-ABS method available on the TIMER2.0 website, coupled with a prognostic analysis, we unveiled potential associations between TMEM25 expression and factors such as mast cell activation, NK cell resting, and T cell regulation. Furthermore, our investigations unveiled associations between TMEM25 and various pivotal immune checkpoints, immunosuppressive agents, and MHC molecules. These results are based on the TISIDB website, which is a multifunctional platform integrating gene and tumour immune system interactions. Additionally, we leveraged the SCNA section of TIMER2.0 to dissect the impact of somatic copy number alterations of the TMEM25 gene on tumor infiltration levels.

### Genetic alterations, methylation analysis and drug sensitivity analysis of TMEM25 in ccRCC

The genetic alterations associated with TMEM25 in ccRCC were carefully analysed using ccRCC data accessed through the cBioPortal website, in particular the Firehose Legacy data from TCGA. The analysis allowed us to identify the main types of TMEM25 mutations and their distribution patterns. To establish the relations between TMEM25 expression levels and gene mutations, we leveraged the capabilities of the muTarget platform, which yielded meaningful insights into these relationships.

In our pursuit of understanding the role of TMEM25 methylation in ccRCC, we turned to platforms like UCSC Xena and UALCAN. These resources illuminated the distinct methylation patterns within ccRCCs and their correlation with various clinical characteristics. In addition, findings from the DNMIVD database suggest that DNA methylation levels of TMEM25 can be used as a predictive marker for survival and diagnosis of ccRCC patients.

Intriguingly, we also aimed to measure the differential sensitivity of certain drugs between groups characterized by high and low TMEM25 expression. To achieve this, we harnessed the “pRRophetic” R package, enabling us to compute the respective IC50 values based on the “cgp2016ExprRma” dataset. This analysis facilitated a comparative assessment of drug sensitivity.

### Statistical analysis

The statistical analysis for this study was executed using the R programming language software, version 4.1.2. To examine variations in gene expression between ccRCC tissues and their corresponding normal tissues adjacent to cancer, we employed the Wilcoxon test. In adherence to conventional practice, significance was attributed to results where p<0.05, indicating statistically significant differences.

### Consent

The authors are accountable for all aspects of the work in ensuring that questions related to the accuracy or integrity of any part of the work are appropriately investigated and resolved.

## References

[1] Siegel RL, Miller KD, Fuchs HE, Jemal A. Cancer statistics, 2022. CA Cancer J Clin. 2022; 72:7–33. 10.3322/caac.21708 35020204

[2] Hsieh JJ, Purdue MP, Signoretti S, Swanton C, Albiges L, Schmidinger M, Heng DY, Larkin J, Ficarra V. Renal cell carcinoma. Nat Rev Dis Primers. 2017; 3:17009. 10.1038/nrdp.2017.9 28276433 PMC5936048

[3] Linehan WM, Ricketts CJ. The Cancer Genome Atlas of renal cell carcinoma: findings and clinical implications. Nat Rev Urol. 2019; 16:539–52. 10.1038/s41585-019-0211-5 31278395

[4] Gray RE, Harris GT. Renal Cell Carcinoma: Diagnosis and Management. Am Fam Physician. 2019; 99:179–84. 30702258

[5] Wolf MM, Kimryn Rathmell W, Beckermann KE. Modeling clear cell renal cell carcinoma and therapeutic implications. Oncogene. 2020; 39:3413–26. 10.1038/s41388-020-1234-3 32123314 PMC7194123

[6] Atkins MB, Tannir NM. Current and emerging therapies for first-line treatment of metastatic clear cell renal cell carcinoma. Cancer Treat Rev. 2018; 70:127–37. 10.1016/j.ctrv.2018.07.009 30173085

[7] Makhov P, Joshi S, Ghatalia P, Kutikov A, Uzzo RG, Kolenko VM. Resistance to Systemic Therapies in Clear Cell Renal Cell Carcinoma: Mechanisms and Management Strategies. Mol Cancer Ther. 2018; 17:1355–64. 10.1158/1535-7163.MCT-17-1299 29967214 PMC6034114

[8] Leibovich BC, Lohse CM, Crispen PL, Boorjian SA, Thompson RH, Blute ML, Cheville JC. Histological subtype is an independent predictor of outcome for patients with renal cell carcinoma. J Urol. 2010; 183:1309–15. 10.1016/j.juro.2009.12.035 20171681

[9] Guida A, Sabbatini R, Gibellini L, De Biasi S, Cossarizza A, Porta C. Finding predictive factors for immunotherapy in metastatic renal-cell carcinoma: What are we looking for? Cancer Treat Rev. 2021; 94:102157. 10.1016/j.ctrv.2021.102157 33607461

[10] Foulquier F, Amyere M, Jaeken J, Zeevaert R, Schollen E, Race V, Bammens R, Morelle W, Rosnoblet C, Legrand D, Demaegd D, Buist N, Cheillan D, et al. TMEM165 deficiency causes a congenital disorder of glycosylation. Am J Hum Genet. 2012; 91:15–26. 10.1016/j.ajhg.2012.05.002 22683087 PMC3397274

[11] Malhotra K, Luehrsen KR, Costello LL, Raich TJ, Sim K, Foltz L, Davidson S, Xu H, Chen A, Yamanishi DT, Lindemann GW, Cain CA, Madlansacay MR, et al. Identification of differentially expressed mRNAs in human fetal liver across gestation. Nucleic Acids Res. 1999; 27:839–47. 10.1093/nar/27.3.839 9889281 PMC148255

[12] Zhang S, Dai H, Li W, Wang R, Wu H, Shen M, Hu Y, Xie L, Xing Y. TMEM116 is required for lung cancer cell motility and metastasis through PDK1 signaling pathway. Cell Death Dis. 2021; 12:1086. 10.1038/s41419-021-04369-1 34789718 PMC8599864

[13] Li Y, Guo W, Liu S, Zhang B, Yu BB, Yang B, Kan SL, Feng SQ. Silencing Transmembrane Protein 45B (TNEM45B) Inhibits Proliferation, Invasion, and Tumorigenesis in Osteosarcoma Cells. Oncol Res. 2017; 25:1021–6. 10.3727/096504016X14821477992177 28244852 PMC7841085

[14] Shen K, Yu W, Yu Y, Liu X, Cui X. Knockdown of TMEM45B inhibits cell proliferation and invasion in gastric cancer. Biomed Pharmacother. 2018; 104:576–81. 10.1016/j.biopha.2018.05.016 29803169

[15] Wrzesiński T, Szelag M, Cieślikowski WA, Ida A, Giles R, Zodro E, Szumska J, Poźniak J, Kwias Z, Bluyssen HA, Wesoly J. Expression of pre-selected TMEMs with predicted ER localization as potential classifiers of ccRCC tumors. BMC Cancer. 2015; 15:518. 10.1186/s12885-015-1530-4 26169495 PMC5015219

[16] Shiraishi T, Ikeda K, Tsukada Y, Nishizawa Y, Sasaki T, Ito M, Kojima M, Ishii G, Tsumura R, Saijou S, Koga Y, Yasunaga M, Matsumura Y. High expression of TMEM180, a novel tumour marker, is associated with poor survival in stage III colorectal cancer. BMC Cancer. 2021; 21:302. 10.1186/s12885-021-08046-6 33757462 PMC7989078

[17] Rao J, Wu X, Zhou X, Deng R, Ma Y. TMEM205 Is an Independent Prognostic Factor and Is Associated With Immune Cell Infiltrates in Hepatocellular Carcinoma. Front Genet. 2020; 11:575776. 10.3389/fgene.2020.575776 33193690 PMC7592400

[18] Hrašovec S, Hauptman N, Glavač D, Jelenc F, Ravnik-Glavač M. TMEM25 is a candidate biomarker methylated and down-regulated in colorectal cancer. Dis Markers. 2013; 34:93–104. 10.3233/DMA-120948 23324576 PMC3809969

[19] Doolan P, Clynes M, Kennedy S, Mehta JP, Germano S, Ehrhardt C, Crown J, O’Driscoll L. TMEM25, REPS2 and Meis 1: favourable prognostic and predictive biomarkers for breast cancer. Tumour Biol. 2009; 30:200–9. 10.1159/000239795 19776672

[20] Li Y, Wang Y, Wang H, Zhang L, Ding Y, Chen S, Yang Q, Chen C. [Effects of lncRNA RP11-770J1.3 and TMEM25 expression on paclitaxel resistance in human breast cancer cells]. Zhejiang Da Xue Xue Bao Yi Xue Ban. 2017; 46:364–70. 10.3785/j.issn.1008-9292.2017.08.04 29256224 PMC10396865

[21] Katoh M, Katoh M. Identification and characterization of human TMEM25 and mouse Tmem25 genes *in silico*. Oncol Rep. 2004; 12:429–33. 10.3892/or.12.2.429 15254712

[22] Meng M, Lan T, Tian D, Qin Z, Li Y, Li J, Cao H. Integrative Bioinformatics Analysis Demonstrates the Prognostic Value of Chromatin Accessibility Biomarkers in Clear Cell Renal Cell Carcinoma. Front Oncol. 2021; 11:814396. 10.3389/fonc.2021.814396 34993155 PMC8724435

[23] Lv J, Zhu P, Yang Z, Li M, Zhang X, Cheng J, Chen X, Lu F. PCDH20 functions as a tumour-suppressor gene through antagonizing the Wnt/β-catenin signalling pathway in hepatocellular carcinoma. J Viral Hepat. 2015; 22:201–11. 10.1111/jvh.12265 24910204 PMC4344823

[24] Gajewski TF, Schreiber H, Fu YX. Innate and adaptive immune cells in the tumor microenvironment. Nat Immunol. 2013; 14:1014–22. 10.1038/ni.2703 24048123 PMC4118725

[25] Kopecký O, Lukesová S, Vroblová V, Vokurková D, Morávek P, Safránek H, Hlávková D, Soucek P. Phenotype analysis of tumour-infiltrating lymphocytes and lymphocytes in peripheral blood in patients with renal carcinoma. Acta Medica (Hradec Kralove). 2007; 50:207–12. 10.14712/18059694.2017.84 18254275

[26] Komohara Y, Hasita H, Ohnishi K, Fujiwara Y, Suzu S, Eto M, Takeya M. Macrophage infiltration and its prognostic relevance in clear cell renal cell carcinoma. Cancer Sci. 2011; 102:1424–31. 10.1111/j.1349-7006.2011.01945.x 21453387

[27] Gutwein P, Schramme A, Sinke N, Abdel-Bakky MS, Voss B, Obermüller N, Doberstein K, Koziolek M, Fritzsche F, Johannsen M, Jung K, Schaider H, Altevogt P, et al. Tumoural CXCL16 expression is a novel prognostic marker of longer survival times in renal cell cancer patients. Eur J Cancer. 2009; 45:478–89. 10.1016/j.ejca.2008.10.023 19070478

[28] Amedei A, Prisco D, D’ Elios MM. The use of cytokines and chemokines in the cancer immunotherapy. Recent Pat Anticancer Drug Discov. 2013; 8:126–42. 10.2174/1574892811308020002 22894642

[29] Atretkhany KN, Drutskaya MS, Nedospasov SA, Grivennikov SI, Kuprash DV. Chemokines, cytokines and exosomes help tumors to shape inflammatory microenvironment. Pharmacol Ther. 2016; 168:98–112. 10.1016/j.pharmthera.2016.09.011 27613100

[30] Xu W, Wu Y, Liu W, Anwaier A, Tian X, Su J, Huang H, Wei G, Qu Y, Zhang H, Ye D. Tumor-associated macrophage-derived chemokine CCL5 facilitates the progression and immunosuppressive tumor microenvironment of clear cell renal cell carcinoma. Int J Biol Sci. 2022; 18:4884–900. 10.7150/ijbs.74647 35982911 PMC9379407

[31] Massari F, Santoni M, Ciccarese C, Santini D, Alfieri S, Martignoni G, Brunelli M, Piva F, Berardi R, Montironi R, Porta C, Cascinu S, Tortora G. PD-1 blockade therapy in renal cell carcinoma: current studies and future promises. Cancer Treat Rev. 2015; 41:114–21. 10.1016/j.ctrv.2014.12.013 25586601

[32] Miao D, Margolis CA, Gao W, Voss MH, Li W, Martini DJ, Norton C, Bossé D, Wankowicz SM, Cullen D, Horak C, Wind-Rotolo M, Tracy A, et al. Genomic correlates of response to immune checkpoint therapies in clear cell renal cell carcinoma. Science. 2018; 359:801–6. 10.1126/science.aan5951 29301960 PMC6035749

[33] Klümper N, Ralser DJ, Bawden EG, Landsberg J, Zarbl R, Kristiansen G, Toma M, Ritter M, Hölzel M, Ellinger J, Dietrich D. *LAG3* (*LAG-3*, *CD223*) DNA methylation correlates with LAG3 expression by tumor and immune cells, immune cell infiltration, and overall survival in clear cell renal cell carcinoma. J Immunother Cancer. 2020; 8:e000552. 10.1136/jitc-2020-000552 32234847 PMC7174079

[34] Takamatsu K, Tanaka N, Hakozaki K, Takahashi R, Teranishi Y, Murakami T, Kufukihara R, Niwa N, Mikami S, Shinojima T, Sasaki T, Sato Y, Kume H, et al. Profiling the inhibitory receptors LAG-3, TIM-3, and TIGIT in renal cell carcinoma reveals malignancy. Nat Commun. 2021; 12:5547. 10.1038/s41467-021-25865-0 34545095 PMC8452744

[35] McDermott DF, Huseni MA, Atkins MB, Motzer RJ, Rini BI, Escudier B, Fong L, Joseph RW, Pal SK, Reeves JA, Sznol M, Hainsworth J, Rathmell WK, et al. Clinical activity and molecular correlates of response to atezolizumab alone or in combination with bevacizumab versus sunitinib in renal cell carcinoma. Nat Med. 2018; 24:749–57. 10.1038/s41591-018-0053-3 29867230 PMC6721896

[36] Motzer RJ, Powles T, Burotto M, Escudier B, Bourlon MT, Shah AY, Suárez C, Hamzaj A, Porta C, Hocking CM, Kessler ER, Gurney H, Tomita Y, et al. Nivolumab plus cabozantinib versus sunitinib in first-line treatment for advanced renal cell carcinoma (CheckMate 9ER): long-term follow-up results from an open-label, randomised, phase 3 trial. Lancet Oncol. 2022; 23:888–98. 10.1016/S1470-2045(22)00290-X 35688173 PMC10305087

[37] Motzer RJ, Hutson TE, Cella D, Reeves J, Hawkins R, Guo J, Nathan P, Staehler M, de Souza P, Merchan JR, Boleti E, Fife K, Jin J, et al. Pazopanib versus sunitinib in metastatic renal-cell carcinoma. N Engl J Med. 2013; 369:722–31. 10.1056/NEJMoa1303989 23964934

[38] Nishiyama A, Nakanishi M. Navigating the DNA methylation landscape of cancer. Trends Genet. 2021; 37:1012–27. 10.1016/j.tig.2021.05.002 34120771

[39] Klutstein M, Nejman D, Greenfield R, Cedar H. DNA Methylation in Cancer and Aging. Cancer Res. 2016; 76:3446–50. 10.1158/0008-5472.CAN-15-3278 27256564

[40] Yang W, Zhou J, Zhang Z, Zhang K, Xu Y, Li L, Cai L, Gong Y, Gong K. Downregulation of lncRNA APCDD1L-AS1 due to DNA hypermethylation and loss of VHL protein expression promotes the progression of clear cell renal cell carcinoma. Int J Biol Sci. 2022; 18:2583–96. 10.7150/ijbs.71519 35414787 PMC8990466

[41] Yang W, Zhang K, Li L, Xu Y, Ma K, Xie H, Zhou J, Cai L, Gong Y, Gong K. Downregulation of lncRNA ZNF582-AS1 due to DNA hypermethylation promotes clear cell renal cell carcinoma growth and metastasis by regulating the N(6)-methyladenosine modification of MT-RNR1. J Exp Clin Cancer Res. 2021; 40:92. 10.1186/s13046-021-01889-8 33691743 PMC7945252

[42] Martincorena I, Campbell PJ. Somatic mutation in cancer and normal cells. Science. 2015; 349:1483–9. 10.1126/science.aab4082 26404825

[43] Jonasch E, Walker CL, Rathmell WK. Clear cell renal cell carcinoma ontogeny and mechanisms of lethality. Nat Rev Nephrol. 2021; 17:245–61. 10.1038/s41581-020-00359-2 33144689 PMC8172121

[44] Peña-Llopis S, Vega-Rubín-de-Celis S, Liao A, Leng N, Pavía-Jiménez A, Wang S, Yamasaki T, Zhrebker L, Sivanand S, Spence P, Kinch L, Hambuch T, Jain S, et al. BAP1 loss defines a new class of renal cell carcinoma. Nat Genet. 2012; 44:751–9. 10.1038/ng.2323 22683710 PMC3788680

[45] Gallan AJ, Parilla M, Segal J, Ritterhouse L, Antic T. BAP1-Mutated Clear Cell Renal Cell Carcinoma. Am J Clin Pathol. 2021; 155:718–28. 10.1093/ajcp/aqaa176 33210135

[46] Cancer Genome Atlas Research Network. Comprehensive molecular characterization of clear cell renal cell carcinoma. Nature. 2013; 499:43–9. 10.1038/nature12222 23792563 PMC3771322

[47] Barrett T, Wilhite SE, Ledoux P, Evangelista C, Kim IF, Tomashevsky M, Marshall KA, Phillippy KH, Sherman PM, Holko M, Yefanov A, Lee H, Zhang N, et al. NCBI GEO: archive for functional genomics data sets--update. Nucleic Acids Res. 2013; 41:D991–5. 10.1093/nar/gks1193 23193258 PMC3531084

[48] Wozniak MB, Le Calvez-Kelm F, Abedi-Ardekani B, Byrnes G, Durand G, Carreira C, Michelon J, Janout V, Holcatova I, Foretova L, Brisuda A, Lesueur F, McKay J, et al. Integrative genome-wide gene expression profiling of clear cell renal cell carcinoma in Czech Republic and in the United States. PLoS One. 2013; 8:e57886. 10.1371/journal.pone.0057886 23526956 PMC3589490

[49] Eckel-Passow JE, Serie DJ, Bot BM, Joseph RW, Cheville JC, Parker AS. ANKS1B is a smoking-related molecular alteration in clear cell renal cell carcinoma. BMC Urol. 2014; 14:14. 10.1186/1471-2490-14-14 24479813 PMC3944917

[50] Karlsson M, Zhang C, Méar L, Zhong W, Digre A, Katona B, Sjöstedt E, Butler L, Odeberg J, Dusart P, Edfors F, Oksvold P, von Feilitzen K, et al. A single-cell type transcriptomics map of human tissues. Sci Adv. 2021; 7:eabh2169. 10.1126/sciadv.abh2169 34321199 PMC8318366

